# *N*-(1-Naphth­yl)-10*H*-9-oxa-1,3-diaza­anthracen-4-amine

**DOI:** 10.1107/S1600536809004693

**Published:** 2009-02-13

**Authors:** Hoong-Kun Fun, Suchada Chantrapromma, Sankappa Rai, Prakash Shetty, Arun M. Isloor

**Affiliations:** a X-ray Crystallography Unit, School of Physics, Universiti Sains Malaysia, 11800 USM, Penang, Malaysia; b Crystal Materials Research Unit, Department of Chemistry, Faculty of Science, Prince of Songkla University, Hat-Yai, Songkhla 90112, Thailand; c Syngene International Ltd, Biocon Park, Plot Nos. 2 and 3, Bommasandra 4th Phase, Jigani Link Road, Bangalore 560 100, India; d Department of Printing, Manipal Institute of Technology, Manipal 576 104, India; e Department of Chemistry, National Institute of Technology-Karnataka, Surathkal, Mangalore 575 025, India

## Abstract

In the mol­ecule of the title compound, C_21_H_15_N_3_O, the 10*H*-9-oxa-1,3-diaza­anthracene ring system is slightly bent, with dihedral angles of 3.99 (6) and 4.80 (6)° between the pyran ring and the pyrimidine and benzene rings, respectively. This ring system makes a dihedral angle of 85.23 (3)° with the naphthalene plane. In the crystal packing, mol­ecules are linked by N—H⋯N hydrogen bonds into chains along the *a* axis and these chains are stacked along the *b* axis. The crystal is further stabilized by weak C—H⋯N and C—H⋯π inter­actions.

## Related literature

For values of bond lengths, see Allen *et al.* (1987 ▶). For background to the bioactivity and applications of naphthyrimidines, see, for example: Bedard *et al.* (2000 ▶); Bohme & Haake (1976 ▶); Erian (1993 ▶); Falardeau *et al.* (2000 ▶); Martinez & Marco (1997 ▶); Tandon *et al.* (1991 ▶); Taylor & McKillop (1970 ▶). For the stability of the temperature controller, see Cosier & Glazer (1986 ▶).
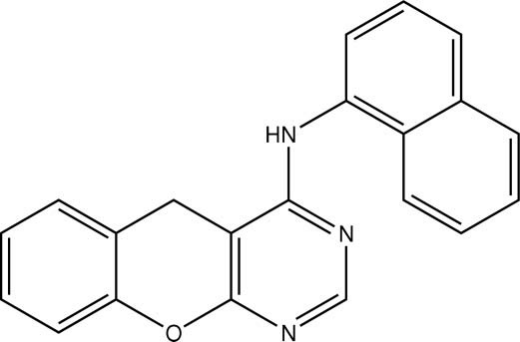


## Experimental

### 

#### Crystal data

C_21_H_15_N_3_O
*M* *_r_* = 325.36
Orthorhombic, 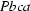

*a* = 13.2762 (3) Å
*b* = 8.8700 (2) Å
*c* = 27.1997 (5) Å
*V* = 3203.03 (12) Å^3^
*Z* = 8
Mo *K*α radiation
μ = 0.09 mm^−1^
*T* = 100 K
0.57 × 0.38 × 0.03 mm

#### Data collection

Bruker SMART APEXII CCD area-detector diffractometer
Absorption correction: multi-scan (*SADABS*; Bruker, 2005 ▶) *T*_min_ = 0.901, *T*_max_ = 0.997
27104 measured reflections
4673 independent reflections
3649 reflections with *I* > 2σ(*I*)
*R*_int_ = 0.042

#### Refinement

*R*[*F* ^2^ > 2σ(*F* ^2^)] = 0.045
*wR*(*F* ^2^) = 0.142
*S* = 1.08
4673 reflections
230 parameters
H atoms treated by a mixture of independent and constrained refinement
Δρ_max_ = 0.35 e Å^−3^
Δρ_min_ = −0.33 e Å^−3^

### 

Data collection: *APEX2* (Bruker, 2005 ▶); cell refinement: *APEX2*; data reduction: *SAINT* (Bruker, 2005 ▶); program(s) used to solve structure: *SHELXTL* (Sheldrick, 2008 ▶); program(s) used to refine structure: *SHELXTL*; molecular graphics: *SHELXTL*; software used to prepare material for publication: *SHELXTL* and *PLATON* (Spek, 2003 ▶).

## Supplementary Material

Crystal structure: contains datablocks global, I. DOI: 10.1107/S1600536809004693/sj2571sup1.cif

Structure factors: contains datablocks I. DOI: 10.1107/S1600536809004693/sj2571Isup2.hkl

Additional supplementary materials:  crystallographic information; 3D view; checkCIF report

## Figures and Tables

**Table 1:** Hydrogen-bond geometry (Å, °) *Cg*1 and *Cg*2 are the centroids of the C1–C3/C11/N1/N2 and C4–C9 rings, respectively.

*D*—H⋯*A*	*D*—H	H⋯*A*	*D*⋯*A*	*D*—H⋯*A*
N3—H1N3⋯N2^i^	0.913 (16)	2.143 (16)	2.9722 (13)	150.6 (14)
C13—H13*A*⋯N2^ii^	0.93	2.62	3.4791 (16)	154
C20—H20*A*⋯N3	0.93	2.60	2.9077 (15)	100
C20—H20*A*⋯N1^iii^	0.93	2.48	3.3232 (17)	150
C10—H10*A*⋯*Cg*1^iii^	0.97	2.76	3.5855 (14)	143
C10—H10*B*⋯*Cg*2^ii^	0.97	2.96	3.6792 (14)	132
C13—H13*A*⋯*Cg*1^ii^	0.93	2.63	3.3503 (14)	135

Symmetry codes: (i) 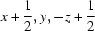; (ii) 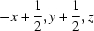; (iii) 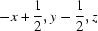.

**Fig. 1.**
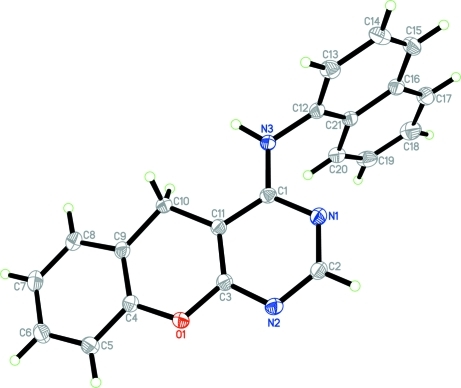
The structure of (I), showing 50% probability displacement ellipsoids and the atom-numbering scheme.

**Fig. 2.**
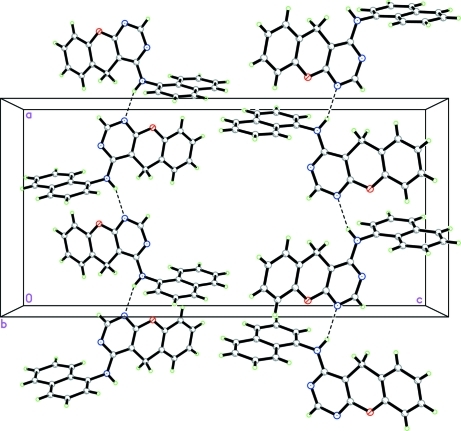
The packing diagram of (I), viewed along the b axis, showing molecular chains along the a axis. Hydrogen bonds are shown as dashed lines.

## supplementary crystallographic information

### Comment

Condensed heterocyclic systems are of considerable interest not only because of their potential biological activity but also because of their versatility as synthons in organic transformations (Bohme & Haake, 1976; Taylor & McKillop, 1970; Erian, 1993). A series of 1,6-naphthyrimidines have been demonstrated to possess antihuman cytomegalovirus (HCMV) activity (Falardeau *et al.*, 2000; Bedard *et al.*, 2000). Furthermore, chromenes and their fused heterocyclic derivatives have attracted a great deal of interest due to their wide applications in the field of pharmaceuticals (Martinez & Marco, 1997; Tandon *et al.*, 1991). In view of these observations, we have synthesized title compound which is a new chromenopyrimidine molecule and its crystal structure was reported here.

In the structure of the title compound (I) (Fig. 1), the 10*H*-9-oxa-1,3-diaza-anthracene ring system is slightly bent as indicated by the dihedral angles between the central ring and the two side rings being 3.99 (6)° (for the C1–C3/C11/N1–N2 ring) and 4.80 (6)° (for the C4–C9 ring). The naphthalene ring system is planar with the largest deviation 0.027 (1) Å for atom C9. The 10*H*-9-oxa-1,3-diaza-anthracene ring is almost perpendicular with the naphthalene ring as shown by the dihedral angle between these two ring systems being 83.00 (15)°. The naphthalene–amine moiety (N3/C12–C21) is in (+)-*syn*-clinal with respect to the C1–C3/C11/N1–N2 ring with a torsion angle C1—N3—C12–C21 = 83.00 (15)°. The bond distances have normal values (Allen *et al.*, 1987).

In the crystal packing, N—H···N hydrogen bonds (Table 1, Fig. 2) link the molecules into chains along the *a* axis and these molecular chains are stacked along the *b* axis. The crystal is further stabilized by weak C—H···N and C—H···π interactions; *Cg*1 and *Cg*2 are the centroids of C1–C3/C11/N1–N2 and C4–C9 rings, respectively (Table 1).

### Experimental

The title compound was obtained by vigorously stirring a solution of 2-amino-4*H*-chromene-3-carbonitrile 0.5 g (2.8 mmol) in 10 ml of dimethyl formamide dimethylacetal which has been heated to reflux for 2 h. Excess dimethyl formamide dimethyl acetal was removed and the residue obtained was dissolved in acetic acid (10 ml). Amine 0.41 g (2.8 mmol) was then added and heated to reflux for an additional 2 h. The reaction mixture was concentrated and finally the residue was purified by column chromatography using petroleum ether–ethyl acetate (60:40 v/v) to get desired compound as a crystalline solid 0.59 g (Yield 63%, m.p. 513–515 K).

### Refinement

The amine H atom was located in a difference map and refined isotropically. The remaining H atoms were placed in calculated positions with d(C—H) = 0.93 Å, *U*_iso_ = 1.2*U*_eq_(C) for aromatic and 0.97 Å, *U*_iso_ = 1.2*U*_eq_(C) for CH_2_. The highest residual electron density peak is located at 0.58 Å from C18 and the deepest hole is located at 0.69 Å from C3.

### Crystal data

**Table:**

C_21_H_15_N_3_O	*D*_x_ = 1.349 Mg m^−^^3^
*M**_r_* = 325.36	Melting point = 513–515 K
Orthorhombic, *P**b**c**a*	Mo *K*α radiation, λ = 0.71073 Å
Hall symbol: -P 2ac 2ab	Cell parameters from 4673 reflections
*a* = 13.2762 (3) Å	θ = 1.5–30.0°
*b* = 8.8700 (2) Å	µ = 0.09 mm^−^^1^
*c* = 27.1997 (5) Å	*T* = 100 K
*V* = 3203.03 (12) Å^3^	Plate, colourless
*Z* = 8	0.57 × 0.38 × 0.03 mm
*F*(000) = 1360	

### Data collection

**Table:**

Bruker SMART APEXII CCD area-detector diffractometer	4673 independent reflections
Radiation source: fine-focus sealed tube	3649 reflections with *I* > 2σ(*I*)
graphite	*R*_int_ = 0.042
Detector resolution: 8.33 pixels mm^-1^	θ_max_ = 30.0°, θ_min_ = 1.5°
ω scans	*h* = −18→18
Absorption correction: multi-scan (*SADABS*; Bruker, 2005)	*k* = −12→12
*T*_min_ = 0.901, *T*_max_ = 0.997	*l* = −37→38
27104 measured reflections	

### Refinement

**Table:**

Refinement on *F*^2^	Primary atom site location: structure-invariant direct methods
Least-squares matrix: full	Secondary atom site location: difference Fourier map
*R*[*F*^2^ > 2σ(*F*^2^)] = 0.045	Hydrogen site location: inferred from neighbouring sites
*wR*(*F*^2^) = 0.142	H atoms treated by a mixture of independent and constrained refinement
*S* = 1.08	*w* = 1/[σ^2^(*F*_o_^2^) + (0.0844*P*)^2^ + 0.3525*P*] where *P* = (*F*_o_^2^ + 2*F*_c_^2^)/3
4673 reflections	(Δ/σ)_max_ = 0.001
230 parameters	Δρ_max_ = 0.35 e Å^−^^3^
0 restraints	Δρ_min_ = −0.33 e Å^−^^3^

### Special details

**Table:**

Experimental. The crystal was placed in the cold stream of an Oxford Cryosystems Cobra open-flow nitrogen cryostat (Cosier & Glazer, 1986) operating at 100.0 (1) K.
Geometry. All e.s.d.'s (except the e.s.d. in the dihedral angle between two l.s. planes) are estimated using the full covariance matrix. The cell e.s.d.'s are taken into account individually in the estimation of e.s.d.'s in distances, angles and torsion angles; correlations between e.s.d.'s in cell parameters are only used when they are defined by crystal symmetry. An approximate (isotropic) treatment of cell e.s.d.'s is used for estimating e.s.d.'s involving l.s. planes.
Refinement. Refinement of *F*^2^ against ALL reflections. The weighted *R*-factor *wR* and goodness of fit *S* are based on *F*^2^, conventional *R*-factors *R* are based on *F*, with *F* set to zero for negative *F*^2^. The threshold expression of *F*^2^ > σ(*F*^2^) is used only for calculating *R*-factors(gt) *etc*. and is not relevant to the choice of reflections for refinement. *R*-factors based on *F*^2^ are statistically about twice as large as those based on *F*, and *R*- factors based on ALL data will be even larger.

### Fractional atomic coordinates and isotropic or equivalent isotropic displacement parameters (Å^2^)

**Table:**

	*x*	*y*	*z*	*U*_iso_*/*U*_eq_	
O1	0.08414 (6)	0.31457 (10)	0.18202 (3)	0.0193 (2)	
N1	0.18466 (7)	0.51429 (12)	0.30718 (4)	0.0183 (2)	
N2	0.05453 (7)	0.44680 (12)	0.25056 (3)	0.0180 (2)	
N3	0.35056 (7)	0.44664 (13)	0.29448 (3)	0.0182 (2)	
H1N3	0.4016 (12)	0.4226 (19)	0.2734 (6)	0.034 (4)*	
C1	0.25345 (8)	0.44337 (14)	0.27880 (4)	0.0156 (2)	
C2	0.09044 (9)	0.51210 (15)	0.29118 (4)	0.0184 (2)	
H2A	0.0435	0.5622	0.3106	0.022*	
C3	0.12498 (8)	0.37767 (14)	0.22322 (4)	0.0163 (2)	
C4	0.14513 (9)	0.22381 (14)	0.15270 (4)	0.0174 (2)	
C5	0.09574 (9)	0.15384 (16)	0.11364 (4)	0.0220 (3)	
H5A	0.0277	0.1716	0.1081	0.026*	
C6	0.14946 (10)	0.05735 (16)	0.08316 (4)	0.0245 (3)	
H6A	0.1175	0.0100	0.0569	0.029*	
C7	0.25153 (10)	0.03159 (16)	0.09199 (4)	0.0234 (3)	
H7A	0.2873	−0.0352	0.0723	0.028*	
C8	0.29956 (9)	0.10591 (15)	0.13028 (4)	0.0202 (3)	
H8A	0.3679	0.0893	0.1355	0.024*	
C9	0.24765 (8)	0.20522 (14)	0.16123 (4)	0.0169 (2)	
C10	0.30044 (8)	0.29292 (15)	0.20116 (4)	0.0178 (2)	
H10A	0.3420	0.2249	0.2204	0.021*	
H10B	0.3442	0.3677	0.1863	0.021*	
C11	0.22622 (8)	0.37036 (14)	0.23449 (4)	0.0156 (2)	
C12	0.37886 (8)	0.52034 (15)	0.33935 (4)	0.0175 (2)	
C13	0.41910 (9)	0.66208 (16)	0.33759 (4)	0.0218 (3)	
H13A	0.4274	0.7097	0.3074	0.026*	
C14	0.44831 (10)	0.73748 (16)	0.38139 (5)	0.0247 (3)	
H14A	0.4748	0.8344	0.3798	0.030*	
C15	0.43757 (9)	0.66800 (16)	0.42576 (5)	0.0240 (3)	
H15A	0.4560	0.7185	0.4543	0.029*	
C16	0.39841 (9)	0.51891 (16)	0.42882 (4)	0.0210 (3)	
C17	0.38629 (10)	0.44401 (18)	0.47457 (4)	0.0273 (3)	
H17A	0.4035	0.4937	0.5035	0.033*	
C18	0.34979 (10)	0.30001 (19)	0.47687 (5)	0.0310 (3)	
H18A	0.3425	0.2527	0.5072	0.037*	
C19	0.32313 (10)	0.22321 (18)	0.43346 (5)	0.0272 (3)	
H19A	0.3001	0.1243	0.4352	0.033*	
C20	0.33080 (9)	0.29311 (16)	0.38844 (4)	0.0215 (3)	
H20A	0.3113	0.2422	0.3601	0.026*	
C21	0.36831 (8)	0.44257 (15)	0.38505 (4)	0.0179 (2)	

### Atomic displacement parameters (Å^2^)

**Table:**

	*U*^11^	*U*^22^	*U*^33^	*U*^12^	*U*^13^	*U*^23^
O1	0.0145 (4)	0.0257 (5)	0.0178 (4)	0.0017 (3)	−0.0020 (3)	−0.0046 (3)
N1	0.0160 (5)	0.0215 (5)	0.0175 (4)	0.0021 (4)	0.0008 (3)	−0.0018 (4)
N2	0.0145 (4)	0.0211 (5)	0.0183 (4)	0.0019 (4)	−0.0010 (3)	0.0001 (4)
N3	0.0128 (4)	0.0267 (6)	0.0152 (4)	0.0008 (4)	−0.0012 (3)	−0.0035 (4)
C1	0.0141 (5)	0.0170 (6)	0.0156 (4)	0.0002 (4)	−0.0003 (4)	0.0016 (4)
C2	0.0163 (5)	0.0212 (6)	0.0178 (5)	0.0019 (5)	0.0020 (4)	−0.0005 (4)
C3	0.0167 (5)	0.0181 (6)	0.0141 (4)	0.0002 (5)	−0.0002 (4)	0.0009 (4)
C4	0.0174 (5)	0.0189 (6)	0.0158 (5)	−0.0008 (5)	0.0009 (4)	0.0005 (4)
C5	0.0202 (6)	0.0272 (7)	0.0186 (5)	−0.0035 (5)	−0.0012 (4)	0.0005 (5)
C6	0.0279 (6)	0.0277 (7)	0.0178 (5)	−0.0051 (5)	−0.0005 (4)	−0.0030 (5)
C7	0.0279 (6)	0.0238 (7)	0.0185 (5)	−0.0003 (5)	0.0052 (5)	−0.0019 (5)
C8	0.0205 (5)	0.0217 (6)	0.0182 (5)	0.0016 (5)	0.0033 (4)	0.0015 (5)
C9	0.0183 (5)	0.0177 (6)	0.0148 (4)	−0.0004 (4)	0.0011 (4)	0.0009 (4)
C10	0.0138 (5)	0.0225 (6)	0.0171 (5)	0.0012 (4)	0.0001 (4)	−0.0019 (4)
C11	0.0152 (5)	0.0173 (6)	0.0144 (4)	0.0008 (4)	−0.0001 (4)	0.0006 (4)
C12	0.0131 (5)	0.0227 (6)	0.0167 (5)	0.0023 (4)	−0.0006 (4)	−0.0028 (4)
C13	0.0198 (6)	0.0243 (7)	0.0213 (5)	0.0001 (5)	−0.0028 (4)	0.0021 (5)
C14	0.0228 (6)	0.0215 (6)	0.0297 (6)	−0.0039 (5)	−0.0053 (5)	−0.0025 (5)
C15	0.0211 (6)	0.0282 (7)	0.0227 (5)	−0.0001 (5)	−0.0047 (4)	−0.0069 (5)
C16	0.0160 (5)	0.0288 (7)	0.0183 (5)	0.0003 (5)	−0.0015 (4)	−0.0033 (5)
C17	0.0223 (6)	0.0427 (9)	0.0169 (5)	−0.0020 (6)	−0.0022 (4)	−0.0014 (5)
C18	0.0262 (6)	0.0453 (9)	0.0214 (6)	−0.0048 (6)	−0.0008 (5)	0.0080 (6)
C19	0.0220 (6)	0.0311 (8)	0.0285 (6)	−0.0060 (6)	−0.0003 (5)	0.0049 (6)
C20	0.0173 (5)	0.0252 (7)	0.0218 (5)	−0.0017 (5)	−0.0003 (4)	−0.0016 (5)
C21	0.0128 (5)	0.0233 (6)	0.0176 (5)	0.0019 (5)	−0.0007 (4)	−0.0026 (5)

### Geometric parameters (Å, °)

**Table:**

O1—C3	1.3649 (13)	C9—C10	1.5085 (16)
O1—C4	1.3927 (14)	C10—C11	1.5050 (15)
N1—C2	1.3245 (15)	C10—H10A	0.9700
N1—C1	1.3511 (14)	C10—H10B	0.9700
N2—C2	1.3355 (15)	C12—C13	1.3669 (19)
N2—C3	1.3432 (15)	C12—C21	1.4287 (16)
N3—C1	1.3582 (14)	C13—C14	1.4202 (17)
N3—C12	1.4345 (14)	C13—H13A	0.9300
N3—H1N3	0.913 (17)	C14—C15	1.3626 (18)
C1—C11	1.4151 (15)	C14—H14A	0.9300
C2—H2A	0.9300	C15—C16	1.4234 (19)
C3—C11	1.3800 (15)	C15—H15A	0.9300
C4—C9	1.3905 (16)	C16—C17	1.4198 (17)
C4—C5	1.3943 (16)	C16—C21	1.4266 (16)
C5—C6	1.3886 (18)	C17—C18	1.368 (2)
C5—H5A	0.9300	C17—H17A	0.9300
C6—C7	1.3951 (19)	C18—C19	1.4082 (19)
C6—H6A	0.9300	C18—H18A	0.9300
C7—C8	1.3878 (17)	C19—C20	1.3764 (17)
C7—H7A	0.9300	C19—H19A	0.9300
C8—C9	1.3999 (16)	C20—C21	1.4191 (19)
C8—H8A	0.9300	C20—H20A	0.9300
			
C3—O1—C4	118.43 (9)	C11—C10—H10B	109.3
C2—N1—C1	116.35 (10)	C9—C10—H10B	109.3
C2—N2—C3	114.05 (10)	H10A—C10—H10B	108.0
C1—N3—C12	121.70 (10)	C3—C11—C1	114.62 (10)
C1—N3—H1N3	120.1 (10)	C3—C11—C10	121.68 (10)
C12—N3—H1N3	116.5 (10)	C1—C11—C10	123.68 (10)
N1—C1—N3	116.89 (10)	C13—C12—C21	120.85 (11)
N1—C1—C11	121.79 (10)	C13—C12—N3	119.46 (11)
N3—C1—C11	121.31 (10)	C21—C12—N3	119.64 (11)
N1—C2—N2	127.97 (11)	C12—C13—C14	120.70 (11)
N1—C2—H2A	116.0	C12—C13—H13A	119.6
N2—C2—H2A	116.0	C14—C13—H13A	119.6
N2—C3—O1	111.42 (9)	C15—C14—C13	120.09 (12)
N2—C3—C11	125.21 (10)	C15—C14—H14A	120.0
O1—C3—C11	123.37 (10)	C13—C14—H14A	120.0
C9—C4—O1	122.82 (10)	C14—C15—C16	120.68 (11)
C9—C4—C5	122.33 (11)	C14—C15—H15A	119.7
O1—C4—C5	114.85 (10)	C16—C15—H15A	119.7
C6—C5—C4	119.18 (11)	C17—C16—C15	121.83 (11)
C6—C5—H5A	120.4	C17—C16—C21	118.52 (12)
C4—C5—H5A	120.4	C15—C16—C21	119.64 (11)
C5—C6—C7	119.81 (11)	C18—C17—C16	121.15 (12)
C5—C6—H6A	120.1	C18—C17—H17A	119.4
C7—C6—H6A	120.1	C16—C17—H17A	119.4
C8—C7—C6	119.84 (12)	C17—C18—C19	120.17 (12)
C8—C7—H7A	120.1	C17—C18—H18A	119.9
C6—C7—H7A	120.1	C19—C18—H18A	119.9
C7—C8—C9	121.60 (11)	C20—C19—C18	120.62 (14)
C7—C8—H8A	119.2	C20—C19—H19A	119.7
C9—C8—H8A	119.2	C18—C19—H19A	119.7
C4—C9—C8	117.14 (10)	C19—C20—C21	120.30 (12)
C4—C9—C10	120.92 (10)	C19—C20—H20A	119.8
C8—C9—C10	121.91 (10)	C21—C20—H20A	119.8
C11—C10—C9	111.40 (9)	C20—C21—C16	119.18 (11)
C11—C10—H10A	109.3	C20—C21—C12	122.82 (11)
C9—C10—H10A	109.3	C16—C21—C12	118.00 (11)
			
C2—N1—C1—N3	179.12 (11)	N1—C1—C11—C3	−0.19 (17)
C2—N1—C1—C11	−0.19 (17)	N3—C1—C11—C3	−179.46 (11)
C12—N3—C1—N1	0.71 (17)	N1—C1—C11—C10	178.62 (11)
C12—N3—C1—C11	−179.98 (11)	N3—C1—C11—C10	−0.66 (18)
C1—N1—C2—N2	0.81 (19)	C9—C10—C11—C3	−10.17 (16)
C3—N2—C2—N1	−0.92 (19)	C9—C10—C11—C1	171.11 (11)
C2—N2—C3—O1	−179.33 (10)	C1—N3—C12—C13	−99.41 (14)
C2—N2—C3—C11	0.44 (18)	C1—N3—C12—C21	83.00 (15)
C4—O1—C3—N2	−172.67 (10)	C21—C12—C13—C14	−2.11 (18)
C4—O1—C3—C11	7.55 (17)	N3—C12—C13—C14	−179.66 (11)
C3—O1—C4—C9	−5.68 (16)	C12—C13—C14—C15	0.8 (2)
C3—O1—C4—C5	174.98 (11)	C13—C14—C15—C16	0.9 (2)
C9—C4—C5—C6	2.60 (19)	C14—C15—C16—C17	−179.90 (12)
O1—C4—C5—C6	−178.05 (11)	C14—C15—C16—C21	−1.21 (18)
C4—C5—C6—C7	0.09 (19)	C15—C16—C17—C18	−179.14 (12)
C5—C6—C7—C8	−1.9 (2)	C21—C16—C17—C18	2.15 (19)
C6—C7—C8—C9	1.13 (19)	C16—C17—C18—C19	−0.2 (2)
O1—C4—C9—C8	177.40 (11)	C17—C18—C19—C20	−1.8 (2)
C5—C4—C9—C8	−3.31 (18)	C18—C19—C20—C21	1.7 (2)
O1—C4—C9—C10	−4.59 (17)	C19—C20—C21—C16	0.33 (18)
C5—C4—C9—C10	174.71 (11)	C19—C20—C21—C12	179.40 (12)
C7—C8—C9—C4	1.42 (18)	C17—C16—C21—C20	−2.21 (17)
C7—C8—C9—C10	−176.57 (12)	C15—C16—C21—C20	179.06 (11)
C4—C9—C10—C11	11.79 (16)	C17—C16—C21—C12	178.68 (11)
C8—C9—C10—C11	−170.29 (11)	C15—C16—C21—C12	−0.06 (17)
N2—C3—C11—C1	0.05 (18)	C13—C12—C21—C20	−177.38 (11)
O1—C3—C11—C1	179.80 (10)	N3—C12—C21—C20	0.17 (17)
N2—C3—C11—C10	−178.78 (11)	C13—C12—C21—C16	1.70 (17)
O1—C3—C11—C10	0.97 (18)	N3—C12—C21—C16	179.25 (10)

### Hydrogen-bond geometry (Å, °)

**Table:**

*D*—H···*A*	*D*—H	H···*A*	*D*···*A*	*D*—H···*A*
N3—H1N3···N2^i^	0.913 (16)	2.143 (16)	2.9722 (13)	150.6 (14)
C13—H13A···N2^ii^	0.93	2.62	3.4791 (16)	154
C20—H20A···N3	0.93	2.60	2.9077 (15)	100
C20—H20A···N1^iii^	0.93	2.48	3.3232 (17)	150
C10—H10A···Cg1^iii^	0.97	2.76	3.5855 (14)	143
C10—H10B···Cg2^ii^	0.97	2.96	3.6792 (14)	132
C13—H13A···Cg1^ii^	0.93	2.63	3.3503 (14)	135

Symmetry codes: (i) *x*+1/2, *y*, −*z*+1/2; (ii) −*x*+1/2, *y*+1/2, *z*;  (iii) −*x*+1/2, *y*−1/2, *z*.
